# Extracellular Vesicles from *Fusarium* *graminearum* Contain Protein Effectors Expressed during Infection of Corn

**DOI:** 10.3390/jof7110977

**Published:** 2021-11-17

**Authors:** Donovan Garcia-Ceron, Rohan G. T. Lowe, James A. McKenna, Linda M. Brain, Charlotte S. Dawson, Bethany Clark, Oliver Berkowitz, Pierre Faou, James Whelan, Mark R. Bleackley, Marilyn A. Anderson

**Affiliations:** 1 Department of Biochemistry and Genetics, La Trobe Institute for Molecular Science, La Trobe University, Bundoora 3086, Australia; d.garcia-ceron@latrobe.edu.au (D.G.-C.); j.mckenna@latrobe.edu.au (J.A.M.); 18686311@students.latrobe.edu.au (L.M.B.); csd51@cam.ac.uk (C.S.D.); m.bleackley@latrobe.edu.au (M.R.B.); 2 La Trobe Comprehensive Proteomics Platform, La Trobe Institute for Molecular Science, La Trobe University, Bundoora 3086, Australia; r.lowe@latrobe.edu.au (R.G.T.L.); p.faou@latrobe.edu.au (P.F.); 3 Cambridge Centre for Proteomics, MRC Toxicology Unit, University of Cambridge, Cambridge CB2 1TN, UK; 4 Centre for Crop and Disease Management, School of Molecular and Life Sciences, Curtin University, Bentley 6102, Australia; bethany.clark@curtin.edu.au; 5 Department of Animal, Plant and Soil Science, La Trobe Institute for Agriculture and Food, La Trobe University, Bundoora 3086, Australia; o.berkowitz@latrobe.edu.au (O.B.); j.whelan@latrobe.edu.au (J.W.)

**Keywords:** EVs, fungal extracellular vesicles, fungi, *Fusarium* *graminearum*, protein effectors, unconventional secretion, virulence factors

## Abstract

*Fusarium* *graminearum* (*Fgr*) is a devastating filamentous fungal pathogen that causes diseases in cereals, while producing mycotoxins that are toxic for humans and animals, and render grains unusable. Low efficiency in managing *Fgr* poses a constant need for identifying novel control mechanisms. Evidence that fungal extracellular vesicles (EVs) from pathogenic yeast have a role in human disease led us to question whether this is also true for fungal plant pathogens. We separated EVs from *Fgr* and performed a proteomic analysis to determine if EVs carry proteins with potential roles in pathogenesis. We revealed that protein effectors, which are crucial for fungal virulence, were detected in EV preparations and some of them did not contain predicted secretion signals. Furthermore, a transcriptomic analysis of corn (*Zea* *mays*) plants infected by *Fgr* revealed that the genes of some of the effectors were highly expressed in vivo, suggesting that the *Fgr* EVs are a mechanism for the unconventional secretion of effectors and virulence factors. Our results expand the knowledge on fungal EVs in plant pathogenesis and cross-kingdom communication, and may contribute to the discovery of new antifungals.

## 1. Introduction

The filamentous fungus *Fusarium graminearum* (*Fgr*) is a devastating agricultural pathogen that infects cereals such as wheat, barley, and corn, where in the latter causes a disease known as Fusarium stalk rot that is characterized by low grain yield and premature plant death [1]. It also leads to losses in grain quality due to the accumulation of mycotoxins, which are toxic for humans and animals [2,3]. There is low efficiency in managing Fusarium stalk rot, partly because the interaction between *Fgr* and the corn plant is not well understood [1]. For these reasons, it is important to explore the infection process of *Fgr* to identify new targets for disease control.

Extracellular vesicles (EVs) are cell-derived particles delimited by lipid membranes that vary in size from 30 to 1000 nm in diameter. They are produced by cells from all three domains of life [4] and have different biological functions and cargo, composed mostly of protein, nucleic acids, and carbohydrates. EVs have been identified in more than 20 yeasts and filamentous fungal species, although EVs from human pathogens such as *Candida albicans* [5], and *Cryptococcus neoformans* [6] are the best characterized.

EVs contribute to virulence of fungal pathogens during infection of their hosts [7,8], which led us to the question of whether EVs from filamentous plant pathogens also have an essential role during infection. EVs from the cotton pathogen *Fusarium oxysporum* f. sp. *vasinfectum* caused a hypersensitive response in cotton leaves [9], indicating that EVs do indeed function in fungal–plant interactions, although the molecules involved in these interactions have not been defined.

One key component of plant-fungal interactions is the secretion of protein effectors by the fungus. Fungal effectors suppress the plant immune response and support fungal survival, and while most known effectors are released from cells via the secretion of signals [10], leaderless effectors have been reported [11,12]. The mechanisms of this unconventional secretion have not been defined, but vesicular transport may have a pivotal role [13,14]. Hence, the study of fungal EVs as potential transporters of virulence factors may lead to the discovery of a new class of effectors previously unrecognized by conventional approaches.

In this study we performed a proteome analysis of EVs, secretome, and whole-cell lysate from *Fgr* and used bioinformatic tools to identify molecules in EV samples that may enhance fungal virulence, such as protein effectors. We also analyzed the *Fgr* transcriptome during the infection of corn to determine if EVs may carry proteins with transcripts expressed during infection. In addition, we optimized the growth medium to improve the yield of EVs from *Fgr* cultures.

The *Fgr* EV preparations contained proteins with annotated roles in pathogenesis together with proteins previously reported as effectors, as well as candidate effectors without conventional secretion signals. Some of the effector candidates were enriched compared to the *Fgr* secretome, suggesting that EVs have a role in the unconventional secretion of virulence factors. Furthermore, we discovered that expression of the genes encoding these potential effectors is increased when *Fgr* infects corn plants.

## 2. Materials and Methods

### 2.1. Fungal Cultures

*Fusarium graminearum* (*Fgr*) strain PH-1 was a gift from Dr. Kim Hammond-Kosack (Rothamsted Research, Harpenden, Herts., UK). The culture used for EV collection was prepared by incubating 500 mL of growth medium with 10^4^ spores/mL in a 2-L flask. The medium contained yeast–nitrogen base (YNB) with ammonium sulfate, without amino acids and carbohydrates (6.7 g/L, US Biological Life Sciences, Salem, MA, USA), with added -Leu dropout supplement (0.69 g/L, Takara, Kusatsu, Shiga, Japan), L-leucine (0.076 g/L, Sigma, St. Louis, MO, USA), and L-glutamic acid (0.5 g/L, Sigma). The components were dissolved in ultrapure water, filter-sterilized using a 0.22-µm Steritop (Merck, Kenilworth, NJ, USA) and maintained at 4 °C until use. This growth medium was named “YNB+”. The cultures were incubated for 5 days at 25 °C with 100 rpm agitation. Mycelia were removed with Miracloth and discarded. The culture fluid was filtered using 0.45-µm membrane filters (HAWP, Merck) and concentrated to about 500 µL using 100-kDa MWCO centrifugal filter units (Merck).

### 2.2. Separation of Extracellular Vesicles (EVs)

EVs were separated by size-exclusion chromatography (SEC) as described previously [15]. Briefly, the concentrated supernatant was mixed with the fluorescent lipophilic dye FM5-95 (Thermo Fisher, Waltham, MA, USA) at a concentration of 1.75 µM (5 µL, 0.1 mg/mL), on a rotary incubator for 15 m at room temperature with protection from light. The sample was loaded onto a 20 mL plastic column (Takara) containing 10 mL of Sepharose CL 2B (Sigma) equilibrated with Dulbecco’s phosphate buffered saline (DPBS, Thermo Fisher). Forty-five fractions (approx. 300 µL each) were eluted with DPBS and collected in black microtiter plates with black bottom (Bunzl, London, UK). The fluorescence of the fractions was measured immediately in a SpectraMax M2 plate reader (Molecular Devices, San Jose, CA, USA). Adjacent fractions with consistent positive relative fluorescence units (RFU) above the baseline were pooled and named “EV sample”. The protein concentration of the EV sample was determined with a Qubit4 (Thermo Fisher). The protein content of the unpooled fractions was quantified by microBCA (Thermo Fisher). All samples were frozen in liquid nitrogen and preserved at −80 °C until further use.

### 2.3. Heat-Treatment of Fgr Cultures

Two controls were made to confirm that the separated particles were not an artifact. First, *Fgr* was grown as described above for 5 d at 25 °C. The mycelia were separated and rinsed with 20 mL of sterile DPBS, before they were heated to 90 °C for 18 h. An aliquot of the heat-treated mycelia was plated on half-strength potato dextrose broth (½ PDB) agar to confirm complete cell death. The remaining mycelia were returned to fresh YNB+ and incubated for 5 d at 25 °C with shaking. After incubation, the mycelia were removed and discarded. The culture supernatant was 0.45-µm-filtered and analyzed by nanoparticle tracking analysis (NTA) as described below. It was compared to a 0.45-µm filtrate from a culture that had not been heat-treated, and to uncultured YNB+. A second control was prepared by processing an *Fgr* culture for SEC isolation as described before but mixing the concentrated supernatant with 5 µL of DPBS, instead of FM5–95.

### 2.4. Preparation of Secretomes and Whole-Cell Lysates (WCL)

Secretomes (secreted soluble proteins and unenriched EVs) were obtained by concentrating 50 mL of the 0.45-µm-filtered culture supernatant to 1 mL using 3-kDa MWCO centrifugal units. WCL were prepared by grinding 80 mg of mycelia in 1 mL of DPBS with 70 mg of glass beads (710–1180 µm, Sigma) on a TissueLyser (Qiagen, Venlo, Limburg, Netherlands), with 30 s cycles shaking at a frequency of 30/s, incubating in an ice bath between cycles. The lysate was centrifuged at 21,130× *g* for 5 min at 4 °C and the supernatant retained for further analysis. The protein concentration of the lysate and secretome was determined immediately after collection with a Qubit4. Samples were stored at −80 °C.

### 2.5. Nanoparticle Tracking Analysis (NTA)

The size and concentration of the particles in the SEC fractions was measured using the scatter mode in a ZetaView instrument (Particle Metrix, software 8.05.12 SP1) with a 405-nm laser, which had been calibrated with a solution of 100-nm beads (Thermo Fisher). The concentration of particles in the samples was adjusted to 30–200 particles per frame, using DPBS in a total volume of 1 mL. Samples were diluted and immediately injected into the instrument’s loading chamber. Eleven chamber positions were measured for data acquisition with a camera sensitivity of 80, shutter speed of 100, brightness between 30 and 255, area between 5 and 1000, and minimum trace length of 15. All samples were analyzed at least in duplicate at a temperature of 25 °C.

### 2.6. Transmission-Electron Microscopy (TEM)

Samples were prepared as described in [16]. Five µl of sample were adjusted to a protein concentration of 1 µg/µL using a Qubit4. Imaging was performed on a Jeol JEM-2100 electron microscope operating at 200 kV. Images were processed with Gatan Digital Micrograph, version 2.32.888.0.

### 2.7. Mass Spectrometry (MS)

EV samples, secretome, and cell lysates were prepared from the same culture, and the samples from three biological replicates were analyzed by LC-MS/MS, as described previously [16]. One µg of peptides was injected into an Ultimate 3000 RSLnano UPLC instrument (Thermo Fisher) coupled to a Q-Exactive HF Orbitrap mass spectrometer (Thermo Fisher). A peptide search was performed using MaxQuant 1.6.3.3, with the label-free quantitation (LFQ) function and matched against the *Fgr* proteome from Uniprot (UP000070720, downloaded 09–10–19). The MaxQuant list was processed in R Studio running R 3.6.0 to remove contaminating proteins, proteins with only one matching peptide, or proteins present in only one biological replicate. LFQ intensities were quantile-normalized and missing LFQ values were imputed with the “candidaev” R package [16]. Proteins with a Benjamini–Hochberg-adjusted *p*-value below 0.05 and with a log_2_-(fold change) (Log_2_-FC) above 1 were considered significantly enriched.

### 2.8. Computational Prediction of Effector Proteins in EV Samples

Proteins with predicted transmembrane domains as well as housekeeping and ribosomal proteins were removed from this analysis. The remaining sequences (356) were submitted to EffectorP 2.0 [17] to predict effector-like properties, ApoplastP to predict apoplastic location [18]; SignalP-5.0, PrediSi, and Phobius to predict signal peptides (SP) [19,20,21]; SecretomeP to predict unconventional secretion (mammalian settings) [22]; PredGPI to predict GPI anchoring [23]; and WolfPSORT and Deep Loc-1.0, to predict subcellular location [24,25]. The percentage of cysteine for each protein was calculated manually.

### 2.9. Gene Ontology (GO) Analysis

The reference genome for *Fgr* in a Uniprot (UP000070720, assembly GCA_900044135.1) was loaded into Blast2GO to annotate proteins with GO terms, using the default settings [26]. The resulting list was used as a reference to perform GO analyses on the EV, secretome and cell lysate proteins, using Fisher’s exact test. The “reduce to most specific” function was applied to all analyses.

### 2.10. Maize Leaf Sheath Infection Assay

*Zea mays* cultivar PH17AW, provided by Corteva Agriscience, was employed in this assay. *Fgr* was grown on synthetic nutrient-poor agar (KH_2_PO_4_ 0.1% (*w*/*v*), KNO_3_ 0.1% (*w*/*v*), MgSO_4_·7H_2_O 0.1% (*w*/*v*), glucose 0.02% (*w*/*v*), sucrose 0.02% (*w*/*v*), Bacto agar 1.5% (*w*/*v*)) at 25 °C with a 16:8 h light-dark cycle, under fluorescent lighting (Grolux, Sylvania, Newhaven, Sussex, UK) for about 3 weeks. Macroconidia were filtered through sterile facial tissue to remove hyphae. Corn plants were infected following protocols with modifications [27,28]. Briefly, a 6-mm disc of filter paper (Whatman Grade AA, GE) was soaked in a solution containing 10^6^ conidia/mL and placed over a 1-mm × 2-mm wound in the corn leaf sheath. Four 8-week-old plants were grown in a greenhouse for each treatment (12 infected plants in total), with six wounds per sheath, and a total of 40 lesions per plant. Wounds were covered in plastic sealing wrap immediately after infection, and the paper disc and sealing wrap were removed after 3 days. Plants were randomized and each plant was considered a biological replicate. Infected tissue was harvested at 3, 5, and 7 days post-inoculation (d.p.i.). Fresh sheath tissue surrounding the lesion was discarded, and a 6-mm disc, collected from the center of the lesion, was frozen immediately after collection using liquid nitrogen, lyophilized, and stored at −80 °C.

### 2.11. RNA Extraction from Infected Corn Tissue and Fgr Mycelium

Total RNA was separated from infected corn tissue and from culture-derived mycelia using TRIzol reagent (Thermo Fisher) and treated with Turbo DNA-free DNase (Thermo Fisher). The RNA sample was purified by precipitation and quality was monitored by agarose gel electrophoresis and UV-Vis spectroscopy. The liquid *Fgr* culture for RNA extraction was prepared by inoculating 50 mL of ½ PDB into a 250 mL flask with 5 × 10^5^ conidia/mL, followed by incubation in the dark at 25 °C with 90 rpm of agitation for 48 h. The mycelia were collected by filtration through Miracloth and were washed with ultrapure water before being frozen in liquid nitrogen, lyophilized, and stored at −80 °C. Each 50-mL culture was considered a biological replicate and four biological replicates were prepared. This sample was named “in vitro” for the transcriptome analysis.

### 2.12. Transcriptome Analysis

Libraries were prepared with an Illumina TruSeq Stranded mRNA kit (San Diego, CA, USA), collecting 75-bp single-ended sequence reads in an Illumina NextSeq sequencer using a NextSeq 500/550 high-output V2 kit. Two runs produced 50 to 70 million reads per biological replicate, with four biological replicates being sequenced per treatment. Data quality was assessed with FastQC (Babraham Bioinformatics, Cambridge, Cambridgeshire, UK) and trimmed with TrimGalore (github.com/FelixKrueger/TrimGalore, version 0.4.1 downloaded on 15 July 2017). Sequence reads were mapped to the *Fgr* PH-1 genome (GCA_000240135.3) using Tophat 2.1.0 [29], and gene expression values were calculated using Cufflinks [29]. The average gene expression per sample was expressed as fragments per kilobase of transcript per million of mapped reads (FPKM), and significant differential gene expression was identified using Cuffdiff [29]. The average transcript expressions of the four biological replicates of all samples (in-vitro, 3, 5, and 7 d.p.i.) were compared and transcripts with an adjusted *p*-value below 0.05 were considered to have significant changes in expression.

## 3. Results

### 3.1. Culture Optimization to Improve the Yield of EVs from Fgr

In our initial experiments we grew *Fgr* in ½ PDB broth and attempted to separate EVs using ultracentrifugation (UC) as we described for *Fusarium oxysporum* (*Fov*) [9]. However, the EV yield from *Fgr* was very low and the sample quality was poor. We then grew *Fgr* on Czapek Dox medium and used size-exclusion chromatography (SEC) rather than UC to isolate EVs because this procedure improved the yield and quality of the EVs from *Fov* [15]. However, when *Fgr* was grown in Czapek Dox medium the culture fluid partially obstructed the 100-kDa filters and could not be concentrated to the level required for SEC. Although the separation of EVs from ½ PDB and Czapek Dox was achieved, EVs from *Fgr* had the best quality and yield when using the “YNB+” medium, which uses amino acids rather than sucrose as the carbon source.

**Figure 1:**
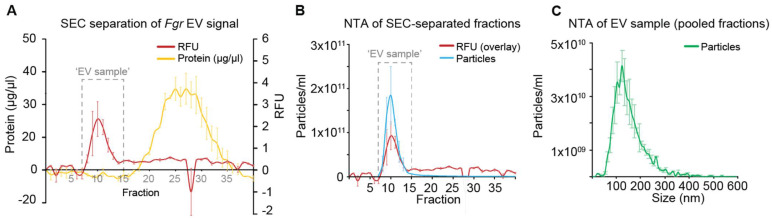
Separation of EVs from *Fusarium graminearum* by size-exclusion chromatography (SEC). (**A**) Particles labeled with the fluorescent dye FM5–95 eluted between fractions 7–15 (red line), while soluble protein eluted between fractions 17 to 35 (yellow line) (n = 2). (**B**) The particle number within each fraction was determined by NTA (blue line, n = 2), and matched the RFU pattern from (**A**). (**C**) The pooled EV sample (fractions 7–15) was analyzed by NTA and had an average of 4.3 × 10^10^ particles/mL of fraction, corresponding to 2.2 × 10^11^ particles/L of culture, and an average particle size of about 120 nm (green line, n = 2). Error bars are SEM.

The elution of the EVs from the SEC column was monitored by fluorescence from the lipid-bound FM5–95 dye (Figure 1A). Fractions 7–15 were thus pooled and named “EV sample”. The fluorescence signal aligned with particle number from the NTA analysis (Figure 1B), which revealed an average particle concentration of 4.3 × 10^10^ particles/mL of pooled fractions (2.2 × 10^11^ particles/L of culture), and an average particle size of about 120 nm (n = 2, Figure 1C). Most of the soluble protein eluted after the EV sample, in fractions 17 to 35 (Figure 1A). The heat-treated *Fgr* cultures and sterile YNB+ medium did not significantly increase the particle number of the EV samples (Figure S1).

**Figure 2:**
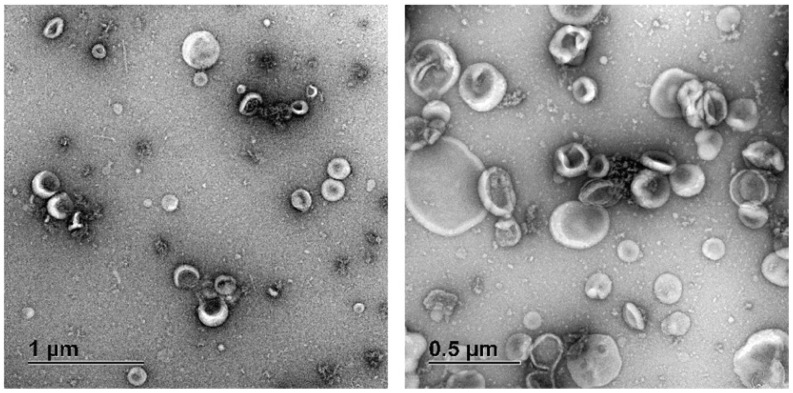
Transmission-electron microscopy (TEM) of EV samples from *Fusarium graminearum*. Five µL of the EV sample were adjusted to 1 µg/µL of protein and were placed on copper grids before treatment with uranyl acetate. TEM revealed spherical structures with apparent sizes ranging from around 50 to 500 nm.

TEM of the EV sample revealed particles partially dehydrated by the uranyl acetate treatment [30], that had the typical cup-like morphology (Figure 2) similar to EVs from other organisms, such as *S. cerevisiae* and *C. albicans* [16,17,18,19,20,21,22,23,24,25,26,27,28,29,30,31,32,33,34,35,36,37,38,39,40,41,42,43,44].

### 3.2. Fgr EV Samples Contain Putative Fungal EV Protein Markers and Proteins with Potential Roles in Toxin Synthesis, Cell Wall Modifications, and Virulence

**Figure 3:**
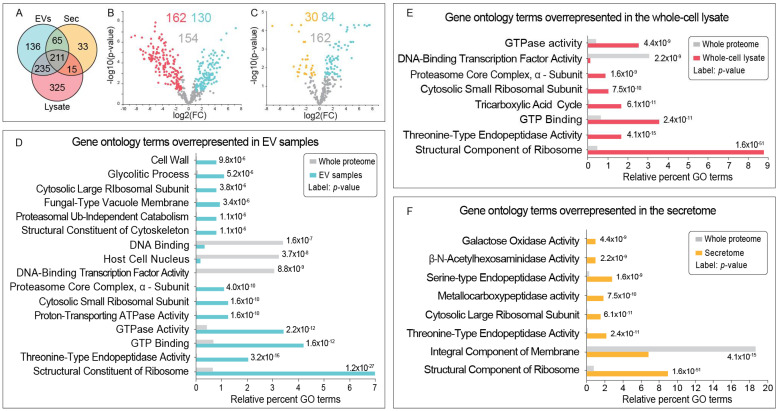
Proteomic analysis revealed potential roles for proteins in the *Fusarium graminearum* (*Fgr*) EV samples. (**A**) Label-free quantitative proteomics detected 647 proteins in the EV samples, 786 in the whole-cell lysate, and 324 in the secretome (Sec). (**B**) Proteins with a *p*-value below 0.05 and a log_2_ fold-change above 1.0 were considered significantly enriched; 130 proteins were enriched in the EV samples (blue) compared with the whole-cell lysate (red); (**C**) 84 proteins in the EV samples (blue) were enriched compared with the secretome (yellow). All gene ontology (GO) comparisons were performed against the complete *Fgr* proteome from Uniprot. GO analysis revealed that proteins in the EV samples (**D**) were overrepresented in cell wall functions and GTPase activity. The cell lysate had more proteins with roles in cellular metabolism or ribosome structure/function (**E**), and the secretome proteins were overrepresented with hydrolase activities (**F**).

The proteomic analysis of the EV samples returned 647 validated proteins (Figure 3A), and 130 of these were enriched in EVs compared to the whole-cell lysate (Table 1, Figure 3B). The five most-abundant proteins in the EV preparations were a subtilisin-like serine protease 6, a polyol transporter, a peptide hydrolase, an AB hydrolase-1 domain-containing protein, and a carboxylic ester hydrolase. From the EV-enriched proteins, 55 were annotated membrane proteins, 28 were involved in transport, 17 were annotated peptidases and nine were involved in carbohydrate hydrolysis. Nine were GTPases and 10 were associated with redox homeostasis. The complete list is available in Table S1.

Gene ontology revealed that GTP-related functions, cell wall and glycolytic processes were overrepresented in the EV proteome, compared to the whole *Fgr* proteome (Figure 3D).

From the 47 putative protein markers reported for *Candida albicans* [16], 16 were detected in *Fgr* EVs. These are similar to *C*. *albicans* proteins, CDC42, FET34, MTS1, orf19.1054, PHR1, RAC1, RHO3, SEC4, SUR7, VAC8, YCK2, YKT6, PHM7, PMA1, SEC61 and YOP1. Three of these were exclusive to EVs (similar to CDC42, RHO3, and YKT6), and eight were enriched in EVs compared to the whole-cell lysate (similar to FET34, PHR1, RAC1, SUR7, YCK2, PHM7, SEC61, and YOP1).

Compared to the whole-cell lysate, 201 proteins were exclusively detected in EVs (Table 2). Some had annotated roles in toxin synthesis such as zearalenone biosynthesis protein 1-like. Other proteins had roles in cell wall modification, such as chitinase 1-like, endo-1,5-alpha-L-arabinanase B-like, glucanase, and mannosidase, and further proteins had roles in virulence, such as effector NIS1-like, and superoxide dismutase.

Proteins of interest that were present in EVs but more abundant in the whole-cell lysate also had potential roles in toxin production, such as core trichothecene cluster protein 8, sirodesmin biosynthesis protein J-like, patulin synthesis protein E-like, aflatoxin biosynthesis protein-like, AF-toxin biosynthesis protein 10-1-like, and penitrem biosynthesis cluster protein S-like. This group of EV proteins also included cell wall-modifying enzymes such as class 3 chitin synthase and chitinase 1-like, and proteins with roles in virulence such as allergen Alt A 7-like, effector SnodProt 1-like, and allergen Fusp4.0101-like.

### 3.3. The Secretome from Fgr Contains Proteins with Potential Roles in Carbohydrate Metabolism, Oxidoreduction and Pathogenesis

Thirty of the 324 proteins detected in the secretome were more abundant in the secretome compared to the EVs (Figure 3C). The five most abundant were a putative endoglucanase, mannitol 2-dehydrogenase, a putative small-secreted cysteine-rich protein (SSCRP), prenyl xanthone synthesis protein C-like, and galactose oxidase. Most of the proteins in the secretomes had annotated roles in metabolism of carbohydrates, hydrolysis, or oxidoreduction (Table S3). The GO analysis revealed that, compared with the complete *Fgr* proteome, more proteins in the secretomes had peptidase functions (Figure 3F). The GO analysis of the whole-cell lysate is also presented (Figure 3E).

### 3.4. EV Samples from Fgr Contain Candidate Protein Effectors

The detection of proteins with roles in fungal virulence in the EV preparations led us to investigate if EVs also transport protein effectors. A computational analysis of proteins in *Fgr* EV samples (Figure S4) revealed 9 effector candidates that have been reported before [31,32], and three proteins that are similar to the known effectors SnodProt1 [33,34], NIS1 [35], and extracellular lipase [36] (Table 3). Our analysis also revealed hydrophobin 3 (FGSG_09066), a previously unreported effector candidate from *Fgr* (Table 4). All these putative effectors had a predicted signal peptide (SP).

Five effector candidates without a predicted SP were also identified: superoxide dismutase [Cu-Zn], chitinase, LysM domain-containing protein, glucoamylase, and glucan endo 1,3-beta-glucosidase eglC-like (Table 4). The *Fgr* superoxide dismutase and chitinase sequences were aligned with characterized sequences from other fungi to determine if the catalytic residues were conserved (Figures S2 and S3, respectively). The complete protein list generated in this analysis is presented in Table S2.

### 3.5. Candidate Protein Effectors Detected in EV Samples Are Expressed In Vivo

**Figure 4:**
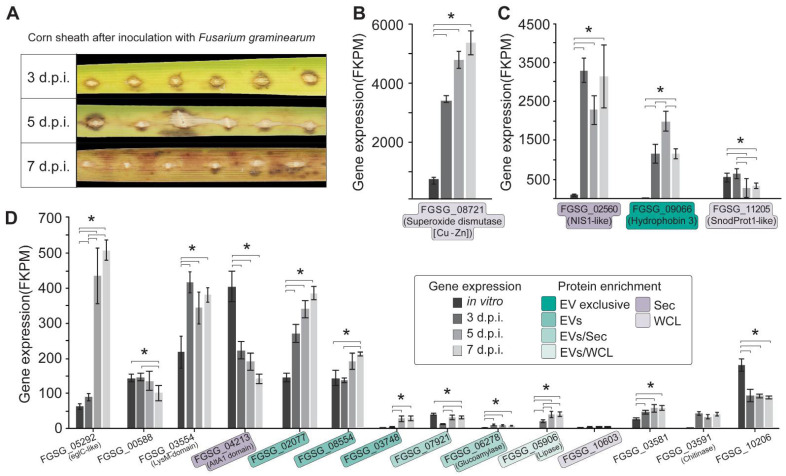
*Fusarium graminearum* (*Fgr*) genes encoding candidate effector proteins detected in extracellular vesicles (EVs) are expressed during corn infection. (**A**) Corn (*Zea mays*) plants were infected with *Fgr* and infected tissue was collected at 3, 5, and 7 days post inoculation (d.p.i.). Mycelia from *Fgr,* grown in vitro, were also collected. Corn tissue and mycelia were analyzed by RNAseq to identify fungal genes expressed during infection and in vitro culture. Gene expression was expressed as fragments per kilobase of transcript per million of mapped reads (FPKM). The gene annotation from the Broad Institute (FGSG_) was employed. Genes from the candidate effectors were significantly expressed in vivo (n = 4). The transcripts were separated by their relative level of gene expression in high (**B**), medium (**C**), and low (**D**). Annotated proteins are labelled, and the rest are hypothetical proteins. (*) significant changes in expression across time points (adjusted *p*-value < 0.05). Error bars are SEM. The protein corresponding to each transcript was retrieved from the EV proteome and its relative abundance is reported in green or purple. Unhighlighted transcripts had no significant differences in protein abundance between EVs, secretome, or cell lysate.

We then asked whether any of these potential effectors are produced during an infection. To determine this, corn plants were inoculated with *Fgr* and tissue samples were taken at 3, 5, and 7 d.p.i. for transcriptome analysis (Figure 4A). The infected corn tissue and *Fgr* mycelium, grown in vitro, returned 14,790 transcripts expressed in the infected corn tissue at one or multiple timepoints. For better interpretation, the genes encoding these transcripts were divided into relatively high (Figure 4B), medium (Figure 4C), and low expression (Figure 4D). Superoxide dismutase [Cu-Zn] transcripts were highly expressed with FKPM values around 5000, while transcripts for secreted effector NIS1-like, hydrophobin 3, and SnodProt1-like had medium expression with FKPM values between 3000 and 500. Transcripts of the uncharacterized effector candidates (Table 3) had relatively low expression (Figure 4D). The statistical analysis of gene expression for individual replicates is presented in Tables S4 and S5, respectively.

## 4. Discussion

The discovery that extracellular vesicles (EVs) from yeast pathogens have a role in the progression of fungal diseases in humans [38,39] led us to examine whether EVs also contribute to the virulence of filamentous fungal pathogens of plants. In this study we isolated EVs from *Fusarium graminearum* (*Fgr*) and searched their proteome for potential virulence factors or effectors that are either transported in EVs through unconventional secretion or are stabilized by EVs in the extracellular environment.

A challenge in the study of EVs from filamentous pathogens has been the preparation of sufficient quantities of EVs of a quality suitable for biochemical analysis. This quality varies between fungal species and the growth medium used for culture [15,40]. We discovered that the culture supernatant from *Fgr* grown in Czapek Dox was viscous and could not be concentrated sufficiently for the separation of EVs by size-exclusion chromatography (SEC). This is likely due to the production of extracellular polysaccharides [41]. Furthermore, we discovered that the use of half-strength potato dextrose broth (½ PDB), which is an undefined medium, produced inconsistencies in the growth of *Fgr* as well as low yields of EVs, impeding further biochemical analyses and compromising experimental reproducibility. To address these issues, we grew *Fgr* in YNB+ medium, which contained amino acids rather than carbohydrates as a carbon source [42]. This solved the viscosity problem and allowed the separation of EVs by SEC.

*Fgr* EVs have been separated before by ultracentrifugation (UC) [43], requiring pooled EVs from several cultures to obtain sufficient material. The authors reported around 4.1 × 10^10^ particles/mL per pooled separation, although the total culture volume required was not reported [45] impeding a direct comparison with our procedure. Our initial EV separations using UC also produced low yields of EVs and poor particle quality. By using SEC and the YNB+ medium, we obtained an average of 2.2 × 10^11^ particles/L of culture (n = 2), with size and morphology consistent with other fungal EVs [9,15,44].

The cargo of EVs from plant pathogens suggests a role for EVs in fungal virulence. For example, *Fusarium oxysporum* EVs contain biosynthetic proteins for secondary metabolites involved in virulence, cell wall-degrading enzymes, and proteases [9,15]. *Fgr* EV samples contained proteins similar to those that produce the toxic secondary metabolites dothistromin, aspirochlorines, solanapyrone, citrinin, and zearalenone [45,46,47,48,49]. The latter is one of the main mycotoxins produced by *Fgr*. The presence of biosynthetic enzymes indicates that EVs may transport phytotoxic secondary metabolites. Additionally, zearalenone, citrinin, and dothistromin have low water solubility [50,51,52], explaining why vesicular transport may facilitate delivery. Interestingly, *Aspergillus parasiticus* employs vesicles to synthesize and release aflatoxin B1 [53].

The characterization of fungal secretomes followed by the prediction of protein effectors is an effective way to identify components of the plant-pathogen interaction [54]. Of the 647 proteins detected in the *Fgr* EV samples in this study, 18 have potential effector properties. Twelve of these have been reported as candidate effectors before [31,32,33,34,35], and six new effector candidates are proposed in this study.

The 12 previously reported candidate effectors had a predicted signal peptide (SP). Indeed, the bioinformatics programs used to identify them selected proteins with conventional effector features, such as SP, high Cys content, and size < 300 a.a. [10]. From these 12 candidates, NIS1, SnodProt1, and extracellular lipase are well characterized effectors in other fungi [33,35,36], while the rest are uncharacterized [31,32].

Our corn infection data revealed that the transcripts from NIS1-like were among the most abundant and had significant differences in expression between the in vitro and in planta samples, implying a role in virulence. Three other genes produced transcripts that increased in abundance as the infection progressed and thus merit further study to evaluate their potential roles in infection. They were FGSG_05295 (eglC-like endoglucanase) and, interestingly, two genes encoding proteins that are enriched in EVs (FGSG_02077 and FGSG_08544).

A promising new effector candidate for *Fgr* is the 82 amino acid-long hydrophobin 3 (FgHyd3) [55]. This protein was detected only in EV samples, had a predicted SP, Cys content of almost 10%, and is predicted to reside in the plant apoplast. In our corn infection study, expression from the encoding gene was almost 400-fold higher in planta than in vitro, suggesting a role in pathogenesis. FgHyd3 is also expressed during the infection of barley by *Fgr* [55]. Germlings from *Fgr* mutants lacking FgHyd3 bind poorly to hydrophobic surfaces, such as plant leaves, and are not as infectious [55]. Other fungal hydrophobins have effector activity [56,57], hence it is possible that FgHyd3 also has effector activity, but this needs to be confirmed experimentally. The observation that FgHyd3 was exclusive to the EV samples suggests that EVs function as an unconventional secretion mechanism for some classes of protein effectors, that EVs could transport FgHyd3 to other areas of the plant, or that EVs shield it from early recognition by the plant’s defenses. Since most of the EV proteomes published to date contain numerous proteins with SP [9,58], we hypothesize that EVs might physically encounter and bind secreted proteins and transport them through the cell wall. Secreted hydrophobic proteins, such as the hydrophobins, may interact with the membrane of EVs and hence be more mobile in the extracellular environment.

The second new effector candidate for *Fgr* is a superoxide dismutase [Cu-Zn] previously named SOD1 (FGSG_08721) [59]. The *Fgr* SOD1 does not have a predicted SP, was enriched in the whole-cell lysate, has 2.2% Cys content and had high expression in our corn infection assay. This supports Yao’s and colleagues observations that SOD1 is highly expressed in *Fgr*-infected wheat coleoptiles, and although they reported it as a cytosolic protein [59], SOD1 from other organisms has been detected extracellularly [60,61]. It is unclear if this occurs exclusively via EVs [60,62]. However, SOD1 secretion has been attributed to EVs [63] and has been detected in the EV proteome from numerous fungi [15,16,44,64,65,66]. SOD1 from *Fgr* and in homologs from *B. cinerea*, *M. oryzae*, *Fusarium* spp., *Verticillium* spp., and the human SOD1 contain a diacidic Asp-Glu motif implicated in unconventional secretion [64]. Hence, we believe that SOD1 may be secreted unconventionally via EVs in *Fgr*, although experimental confirmation is still required.

The remaining group of proposed effectors are carbohydrate-active enzymes (CAZy). One chitinase (FGSG_03591) has similarity to chitinases from other fungal pathogens [67,68]. This *Fgr* chitinase does not have a predicted SP although it is annotated as secreted (Uniprot), has >300 a.a., and has a low Cys content. The gene encoding this chitinase was expressed at relatively low levels during our corn infection assay, and did not change as the infection progressed, indicating that its involvement in virulence may differ from other chitinases that are highly expressed in vivo [69].

Another candidate effector was an eglC-like endoglucanase (FGSG_05292), which in *A. niger* is involved in the degradation of plant cell walls [70]. The role of the eglC-like has not been elucidated in *Fgr*, although our infected corn transcriptome data revealed almost a five-fold increase in gene expression between 3 and 5 d.p.i. Such increase is a characteristic of some effectors [71].

One further effector candidate is a LysM domain-containing protein (FGSG_03554). LysM proteins interact with chitin and support fungal survival [72]. The last candidate effector is a glucoamylase (FGSG_06278) with 57% identity to BcGs1 from *B. cinerea*. BcGs1 causes necrosis, accumulation of ROS, and cell death in different hosts [73]. The *Fgr* glucoamylase and BcGs1 have no predicted SP although they are potentially secreted (WolfPSORT, DeepLoc 1.0), suggesting that they are unconventionally secreted. The transcript expression of this protein during corn infection was low compared with other effector candidates, hence it is possible that the abundance of this glucoamylase does not need to be as high as other candidate effectors, since the expression of fungal effector genes is known to be differentially regulated [74,75].

The variety of CAZy enzymes detected in EV samples from *Fgr* suggests roles on host pathogenesis and EV release. For instance, the endo-1,5-alpha-L-arabinanase B-like is a virulence factor in *B. cinerea* during infection of Arabidopsis [76], and a similar arabinanase B was detected in *Fgr* EV samples (FGSG_11468). The substrate for this arabinanase is yet to be defined. Conversely, the presence of chitinase and glucanase in EV preparations suggests that these enzymes loosen the fungal cell wall and facilitate EV release [77].

The leaderless candidate effectors identified in this study and the ones reported previously [11,12] have characteristics that would prevent their identification by bioinformatic tools, such as lack of SP or low Cys content. This is not a limitation of these tools, but rather an unintended bias towards proteins that fit conventional effector criteria. Our results support evidence that a different class of protein effectors exists that are transported via unconventional secretion mechanisms [78,79], and in the case of *Fgr* this is likely to occur via EVs. The purification of these candidate effectors, their inplanta study, and the generation of knockout fungal strains can confirm this notion.

Yang and colleagues identified 154 secretome proteins from *Fgr* that have potential roles in the pathogenesis of wheat and barley [80]. We detected 21 of these proteins in our secretome data, with the majority having annotated functions as glycosidases, proteases, and esterases.

EVs from *Fgr* contained some of putative EV protein markers that have been reported for *C. albicans*. Among these, the eisosomal SUR7 has similarity to the mammalian tetraspanins, making it one ideal candidate to be a true fungal EV marker [16]. SUR7 has been detected in EVs from *Zymoseptoria tritici* [81], *S. cerevisiae* [46] and *A. fumigatus* [64]. Similarly, *C. neoformans* EVs contain proteins with SUR7 domains [6] suggesting that SUR7 is a conserved protein marker of fungal EVs.

In summary, we demonstrate that the filamentous fungal pathogen *Fusarium graminearum* produces extracellular vesicles, and their cargo includes proteins associated with virulence that are expressed during the infection of corn. Evidence from other fungal EV studies [5,7,9] suggests that EVs from *Fgr* could support the infection of corn, although future efforts must be directed at determining the specific role of these EVs.

These results contribute to the elucidation of the mechanism of action of EVs from plant pathogens, which is mostly unknown, and indicate that EVs are a mechanism for unconventional secretion that could protect and transport secreted proteins with conventional secretion signals. Our study has revealed effector candidates that might be involved in pathogenesis and are of interest for future research.

## Figures and Tables

**Table 1:** Most abundant proteins detected in *Fusarium graminearum* (*Fgr*) EV samples compared to the whole-cell lysate. LFQ-based proteomics revealed 647 proteins in the *Fgr* EV samples. Proteins with a *p*-value below 0.05 and with a log_2_-fold change above 1.0 were considered significantly enriched. The 20 most-abundant proteins are presented. Homologs to uncharacterized proteins were identified with BLAST and the E-value of the best match is reported. Gene ontology (GO) terms were obtained with Blast2GO; P: biological process, C: cellular component, F: molecular function.

Uniprot ID	Protein Name	log_2_FC	GO Terms
I1S3S6 ^1^	Putative subtilisin-like serine protease (E-value: 0.0)	7.17	C: cell wall
I1RJE2	Polyol transporter 5	5.99	P: transmembrane transport
A0A1C3YMP0 ^1^	Peptide hydrolase	5.76	F: aminopeptidase activity
I1RQZ5 ^1^	AB hydrolase-1 domain-containing protein	5.39	
A0A098DKT1 ^1^	Carboxylic ester hydrolase	5.14	F: hydrolase activity
I1RY25	Niemann–Pick type C-related protein 1 (E-value: 0.0)	5.07	C: integral component of membrane
I1RUM2 ^1^	Extracellular protein (E-value: 4.5 × 10^−164^)	4.96	
A0A1C3YIM6 ^1^	Peptidase_M14 domain-containing protein	4.79	F: metallocarboxypeptidase activity
I1S050	Casein kinase I isoform gamma 2	4.70	F: protein serine/threonine kinase
A0A1C3YJM7 ^1^	Amine oxidase	4.67	P: oxidation-reduction process
I1S2H9	Magnesium and cobalt transporter	4.49	C: integral component of membrane
I1RP91	Siderophore iron transporter 1	4.31	P: transmembrane transport
A0A1C3YNA9 ^1^	Putative serine carboxypeptidase	4.30	F: serine-type carboxypeptidase
A0A098DS79 ^1^	Gamma-glutamyltransferase (E-value: 0.0)	4.28	F: glutathione hydrolase activity
V6R949	K(+)/H(+) antiporter 1	4.28	F: solute:proton antiporter activity
A0A098E0Z5	H(+)/Cl(−) exchange transporter 5	4.27	F: voltage-gated Cl channel activity
I1RJ42 ^1^	Alpha-amylase (E-value: 0.0)	4.26	F: alpha-amylase activity
I1RDK3	Flotillin-like protein 1	4.20	
I1RMG9 ^1^	Iron transport multicopper oxidase FET3 precursor	4.11	F: oxidoreductase activity
I1RF73 ^1^	Beta-fructofuranosidase (E-value: 0.0)	4.07	P: carbohydrate metabolic process

^1^ Protein also detected in the secretome.

**Table 2:** Proteins in the *Fusarium graminearum* (*Fgr*) EV samples and not in the whole-cell lysate have putative roles in toxin synthesis, carbohydrate metabolism, hydrolysis, and vesicle transport. LFQ-based proteomics revealed 647 proteins present in the *Fgr* EV samples. From these, 201 proteins were exclusive to EVs compared with the cell lysate. Several proteins had annotated roles in metabolite biosynthesis, carbohydrate metabolism, hydrolysis, and vesicle transport. Gene ontology (GO) terms were obtained with Blast2GO; P: biological process, C: cellular component, F: molecular function.

Uniprot ID	Protein Name	GO Terms
**Host-pathogen interactions**		
A0A1C3YLT0	Allergen Asp f 9-like	F: hydrolase; P: cell wall organization
I1RF56	Rubrofusarin-specific efflux pump aurT	P: transmembrane transport
I1RFS2	Secreted effector NIS1-like	
I1RGY5	Allergen Asp f 9-like	F: hydrolase; P: cell wall organization
I1RIM4 ^1^	Allergen Asp f 34-like	
**Transport**		
A0A1C3YHZ2	GTP-binding protein RHO3-like	F: GTPase activity; F: GTP binding
A0A1C3YJH3	Multidrug resistance protein FNX1	P: transmembrane transport
A0A1C3YK53	VPS74	F: phosphatidylinositol-4-phosphate binding
I1RAS9 ^1^	VPS10-like	P: protein transport
I1RFK0	GTP-binding protein RHY1	F: GTPase activity; F: GTP binding
I1RG99	VPS35	P: endosome to Golgi transport
I1RN81	CDC42Sp-like	F: GTPase activity; F: GTP binding
I1RQD6	SEC17 homolog	P: vesicle-mediated transport
I1S278	Syntaxin PEP12	P: vesicle-mediated transport
I1SAM5	v-SNARE protein VTI1	P: vesicle-mediated transport
**Hydrolysis**		
A0A098DV80 ^1^	Podosporapepsin-like	F: aspartic-type endopeptidase activity
A0A0E0RMK7 ^1^	N-acetyl-beta-glucosaminidase 1-like	P: carbohydrate metabolic process
A0A1C3YMS8 ^1^	Mannanase B	P: carbohydrate metabolic process
I1REC8 ^1^	Probable secreted lipase ARB_02369	F: hydrolase activity
I1RF87 ^1^	Chitinase 1-like	P: carbohydrate metabolic process
I1RHG0 ^1^	Chitinase 1-like	P: carbohydrate metabolic process
I1RHW3	Ribonuclease Trv	F: RNA binding
I1RJF8 ^1^	Oryzapsin B-like	F: aspartic-type endopeptidase activity
I1RLG1 ^1^	Aspartic proteinase yapsin-6-like	F: aspartic-type endopeptidase activity
I1RMU2 ^1^	Laminarinase eglC-like	P: carbohydrate metabolic process
I1RR60 ^1^	Subtilisin protease 6-like	C: cell wall
I1RRY4 ^1^	Endo-1,3(4)-beta-glucanase-like	P: carbohydrate metabolic process
I1RXM5 ^1^	Lipase 4-like	F: hydrolase activity
I1S2W9 ^1^	Carboxypeptidase MCPB-like	F: metallocarboxypeptidase activity
I1S3J9 ^1^	Secreted lipase ARB07186/07185-like	
I1S3S2 ^1^	Endo-1,5-alpha-L-arabinanase B-like	P: xylan catabolic process
V6R5G9	Exo-1,3-beta-glucanase-like	P: carbohydrate metabolic process
V6R5Q6 ^1^	Man(9)-alpha-mannosidase 1b-like	F: mannosyl hydrolysis; C: membrane
**Biosynthesis**		
A0A098DAH0	Yanuthone D synthesis protein D	
A0A098DV37	Pestheic acid biosynthesis cluster protein K-like	P: oxidation-reduction process
A0A098DVT4	Sesquiterpene synthase BOT2	C: membrane; F: lyase activity
A0A1C3YLJ5	Anditomin synthesis protein L-like	C: integral component of membrane
A0A1C3YLR9	Leucinostatins biosynthesis cluster protein R-like	F: phospholipase D activity
A0A1C3YMY7	Aspirochlorine biosynthesis protein Q-like	
I1R9G1 ^1^	Solanapyrone biosynthesis protein 5-like	F: oxidoreductase; F: FAD binding
I1RII9 ^1^	Citrinin synthesis protein MPL7-like	F: oxidoreductase activity
I1RS87	Dothistromin biosynthesis protein epoA-like	F: cis-stilbene-oxide hydrolase activity
I1RT88	Pestheic acid biosynthesis cluster protein L-like	F: oxidoreductase activity
I1RUE8 ^1^	Zearalenone biosynthesis protein 1-like	F: oxidoreductase; F: FAD binding
I1RXR7 ^1^	Terrein biosynthesis cluster protein terF-like	
I1S011	Himeic acid A biosynthesis cluster protein E-like	C: integral component of membrane
I1S1K2	Tropolone synthesis protein G	
I1S6B9 ^1^	Prenyl xanthone synthesis protein C-like	F: oxidoreductase activity

^1^ Protein also detected in the secretome.

**Table 3:** Effector candidates detected in the EV samples from *Fusarium graminearum* (*Fgr*) that have been reported previously. The EV proteome from *Fgr* had 12 effector candidates that have been reported previously, although only seven were identified by EffectorP 2.0. All proteins had a predicted signal peptide (Uniprot). The “Enrichment” column indicates the sample in which a protein was most abundant (Sec up: enriched in secretome; EV up: enriched in EV samples; Not sig: no statistical difference). Effectors with characterized function.

Uniprot ID	Protein Name (Gene Symbol)	Length (a.a.)	Enrichment	EffectorP2	Effector Function
I1RFS2	Effector NIS1-like (FGSG_02560)	140	Sec up	non-effector	cell death [35]
I1S341	SnodProt1-like (FGSG_11205)	140	Not sig	unlikely effector	required for virulence [33,34]
I1RPD9	Extracellular lipase (FGSG_05906) ^1^	349	EV up	effector	inhibits innate immunity [36]
I1RUM2	Hypothetical protein FGSG_07921	221	Not sig	effector	unknown [32]
I1RIV3	Hypothetical protein FGSG_03748	253	EV up	effector	unknown [32]
I1RIE9	Hypothetical protein FGSG_03581	198	Not sig	effector	unknown [31]
I1REI8	Hypothetical protein FGSG_02077	184	EV up	non-effector	unknown [31]
I1RAQ3	Hypothetical protein FGSG_00588	160	Not sig	unlikely effector	unknown [31]
I1RW93	Hypothetical protein FGSG_08554	207	EV up	non-effector	unknown [31]
I1RK25	AltA1 domain-containing protein FGSG_04213	166	Sec up	effector	unknown [31]
I1S0H8	Hypothetical protein FGSG_10206	162	Not sig	effector	unknown [31]
I1S1J8	Hypothetical protein FGSG_10603	158	Not sig	effector	unknown [31]

^1^ Confirmed effector characterized in *F*. *graminearum* [36].

**Table 4:** Prediction of new effector candidates in EV samples from *Fusarium graminearum* (*Fgr*). The computational prediction returned sequences with effector potential with and without predicted signal peptide (SP). Uniprot ID and gene symbol are shown in parenthesis. “Enrichment” indicates if the protein was most abundant in EVs, or secretome, or cell lysate. The EffectorP2.0 result was included to monitor the prediction of candidate effectors with unconventional characteristics (size > 300 a.a., Cys < 2%, no SP). The consensus of PrediSi, Uniprot, SignalP 5.0, and Phobius was used to determine the potential presence of signal peptide. SecretomeP 2.0 was used to predict leader-less secretion under the mammalian settings, where a score > 0.5 indicates possible secretion. PredGPI was used to predict GPI anchoring. ApoplastP 1.0, WolfPSORT and DeepLoc 1.0 were used to predict the cellular location of the candidates.

Effector Candidate	Enrichment	Length (a.a.)	Effector P 2.0	Cys %	Signal Peptide ^1^	Secretome P 2.0	PredGPI	Apoplast P 1.0	Location Prediction
Hydrophobin 3 (I1RXJ5, FGSG_09066)	EV exclusive	82	effector	9.8	yes	NA	unlikely	yes	extracellular/ mitochondria
Superoxide dismutase [Cu-Zn] (A0A098DGQ1, FGSG_08721)	cell lysate	228	effector	2.2	no	0.706	-	no	cytoplasm/ nucleus
Chitinase (I1RIF9, FGSG_03591)	no difference	417	non effector	0.5	no	0.505	-	yes	cytoplasm
LysM domain-containing protein (I1RIC3, FGSG_03554)	no difference	403	non effector	0.2	no	0.747	-	no	cytoplasm/ nucleus
Glucoamylase (A0A1C3YK33, FGSG_06278)	no difference	667	non effector	1.2	no	0.518	-	yes	extracellular
Glucan endo-1,3-beta-glucosidase eglC-like (I1RMU2, FGSG_05292)	no difference	409	non effector	1.2	no	0.703	-	yes	extracellular/ cell membrane

## Data Availability

Proteomics data have been deposited on the ProteomeXchange consortium via the PRIDE repositorium [38] (ID: PXD028657). The archives include RAW files, MaxQuant search parameters and .txt output file, and the *Fgr* reference genome FASTA file. The source code used in this study was adapted from a previous study [17] and is available at github.com/csdawson/*Fgr*ev. The transcriptomics data have been deposited on the sequence read archive database under identifier PRJNA529541.

## Supplementary Materials

The following are available online at https://www.mdpi.com/article/10.3390/jof7110977/s1, Figure S1. Controls for the separation of EVs from *Fusarium graminearum* (*Fgr*) by SEC. Figure S2. The superoxide dismutase [Cu-Zn] (SOD1) from F. *graminearum* (*Fgr*) contains a diacidic amino acid motif implicated in unconventional secretion. Figure S3. Sequence alignment of the chitinase GH18 domain. Figure S4. Computational prediction of effector candidates detected in EV samples from *Fusarium graminearum* (*Fgr*). Table S1. List of proteins detected in EVs from *Fusarium graminearum* (*Fgr*). Table S2. List of proteins employed in the computational effector prediction analysis. Table S3. Proteins identified in the secretome from *Fusarium graminearum* (*Fgr*). Table S4. List of transcripts identified in corn (*Zea mays*) infected by *Fusarium graminearum* (*Fgr*). Table S5. Gene expression values per biological replicate.
