# Supplementary material for: Extracellular Vesicles from Fusarium graminearum Contain Protein Effectors Expressed during Infection of Corn
Source: J Fungi (Basel). 2021 Nov 17;7(11):977. doi: 10.3390/jof7110977 (PMC8625442; doi:10.3390/jof7110977)
Supplement: Supplementary file 1 [file jof-07-00977-s001.zip › 6.Supporting information - Figures.pdf]

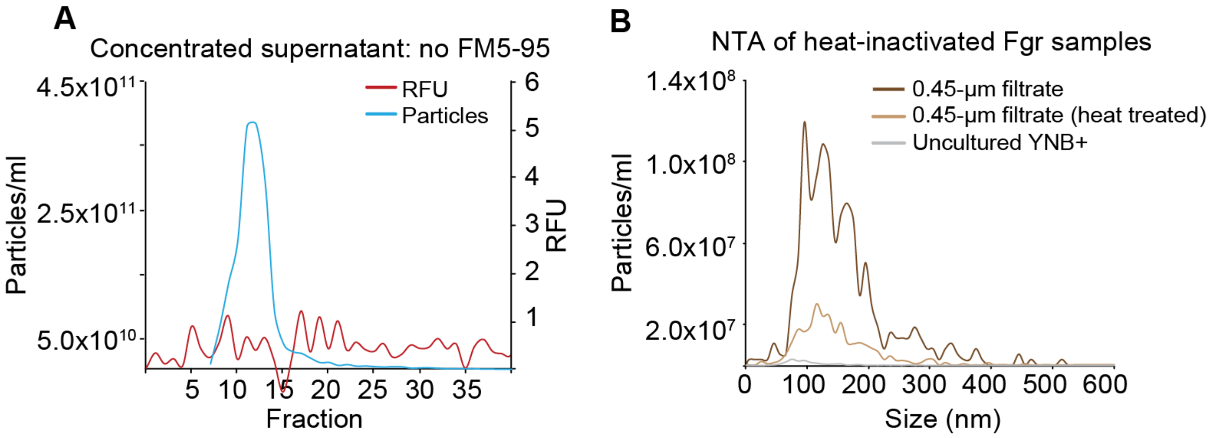

Supplementary Figure S 1. Controls for the separation of EVs from *Fusarium graminearum* (*Fgr*) by SEC. (**A**) An Fgr culture was processed for EV separation and mixed with DPBS instead of FM5-95 before SEC. Fluorescence of all fractions was recorded (red line) and their particle number determined by NTA (blue line) (n=1). (**B**) Fgr was grown for 5 d, then the mycelia were heat-treated before being transferred to fresh medium that was incubated for 5 d. NTA detected 4.1x1010 particles/L in the 0.45-µm filtrate of the heat-treated Fgr culture, 4.8X1010 particles/L in the untreated 0.45 µm filtrate, and 1.9X1010 particles/L in the sterile YNB+ (n=1).

```
Human SOD1      1 ------------------------------------------------------------
Fgr SOD1        1 MRAEGSADNEGVQSRSWWKAPCLVPSEARVSRCFGPRFLFKTPLSSPLHLHHSILNCFIP
Yeast SOD1      1 ------------------------------------------------------------

Human SOD1      1 -------------MATKAVCVLKGDGPVQGIINFEQKESNGPVKV-WGSIKGLTEGLHGF
Fgr SOD1       61 LPKFPRNKNKKTVKMVKAVSVLRGDSKVSGTVVFEQESESAPTTITWDITGNDPNAKRGF
Yeast SOD1      1 --------------MVQAVAVLKGDAGVSGVVKFEQASESEPTTVSYEIAGNSPNAERGF
                                 .:**.**:**. *.* : *** ... *..: :    .  :. :**
Human SOD1     47 HVHEFGDNTAGCTSAGPHFNPLSRKHGGPKDEERHVGDLGNVTADKDGVADVSIEDSVIS
Fgr SOD1      121 HIHTFGDNTNGCTSAGPHFNPHNKTHGAPSDETRHVGDLGNVETDGQGNAKGSVTDSLIK
Yeast SOD1     47 HIHEFGDATNGCVSAGPHFNPFKKTHGAPTDEVRHVGDMGNVKTDENGVAKGSFKDSLIK
                  *:* *** * **.******** .:.**.*.** *****:*** :* :* *. *. **:*.

Human SOD1    107 LSGDHCIIGRTLVVHEKADDLGKGGNEESTKTGNAGSRLACGVIGIAQ
Fgr SOD1      181 LIGPHSVIGRTVVIHAGTDDLGKGDGEESLKTGNAGPRPACGVIGISN
Yeast SOD1    107 LIGPTSVVGRSVVIHAGQDDLGKGDTEESLKTGNAGPRPACGVIGLTN
                  * *  .::**::*:*   ******. *** ****** * ******:::
```

Supplementary Figure S 2. The superoxide dismutase [Cu-Zn] (SOD1) from *Fusarium graminearum* (*Fgr*) contains a diacidic amino acid motif implicated in unconventional secretion. The computational prediction of putative effectors from *Fgr* returned two candidates. One was a SOD1 without a predicted signal peptide, that was interrogated to find links to unconventional protein secretion. Its sequence (*Fgr* SOD1) was aligned with the S. cerevisiae (yeast) and human SOD1 sequences. The *Fgr* SOD1 contains a diacidic Asp-Glu motif (highlighted in red) previously implicated in non-Golgi secretion of SOD1 in *S. cerevisiae* (Cruz-Garcia et al., 2017).

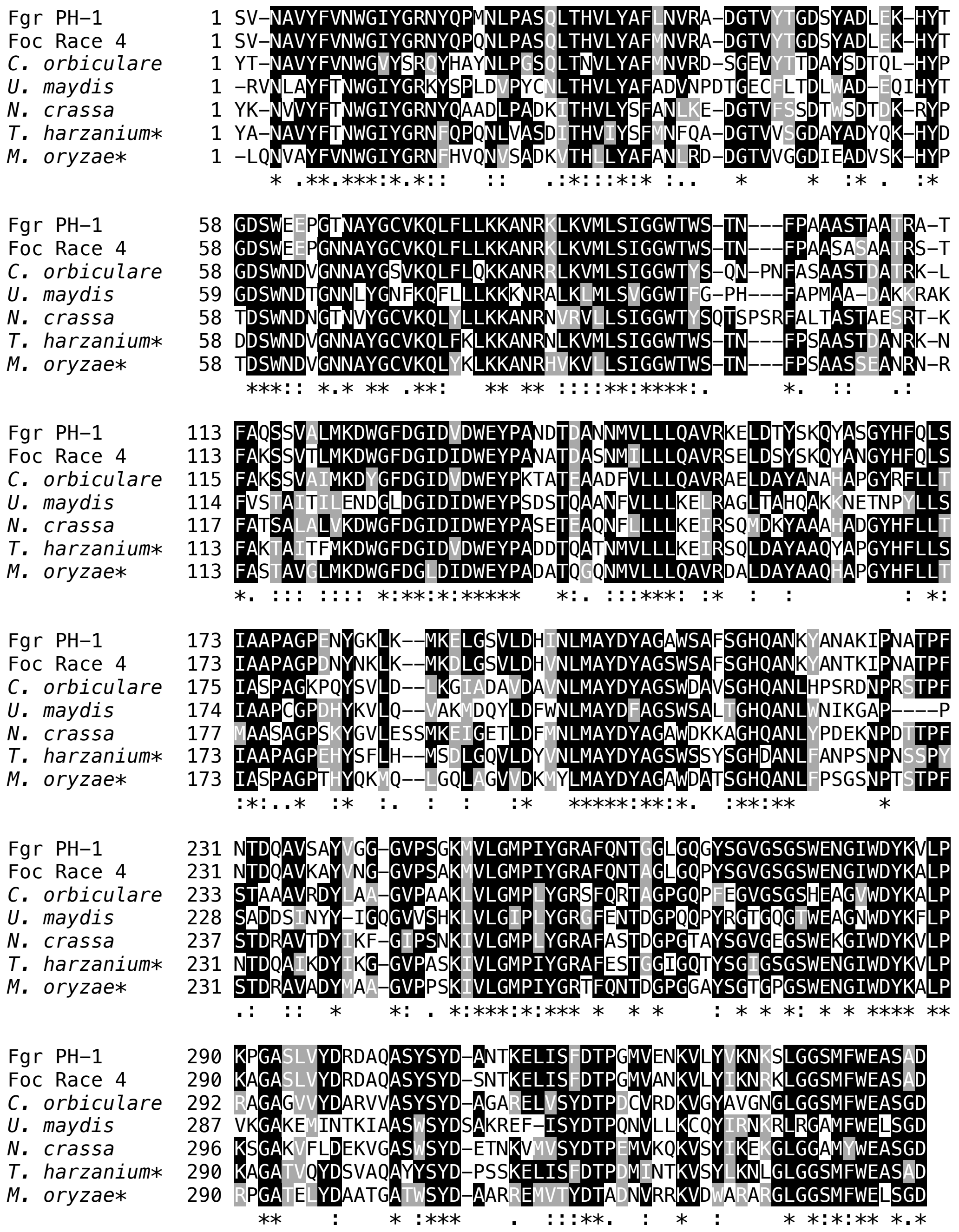

Supplementary Figure S 3. Sequence alignment of the chitinase GH18 domain. The computational effector analysis identified a chitinase from *Fusarium graminearum* (*Fgr*) that was detected in EV samples and shares similarity with chitinases from other fungal pathogens. The Glyco-18 domain from these chitinases was aligned for *Fgr*, *Fusarium oxysporum* f. sp. *cubense* (Foc Race 4), *Ustilago maydis*, *Neurospora crassa*, *Colletotrichum orbiculare*, *Trichoderma harzanium*, and *Magnaporthe oryzae*. *Chitinase sequences with predicted signal peptides.

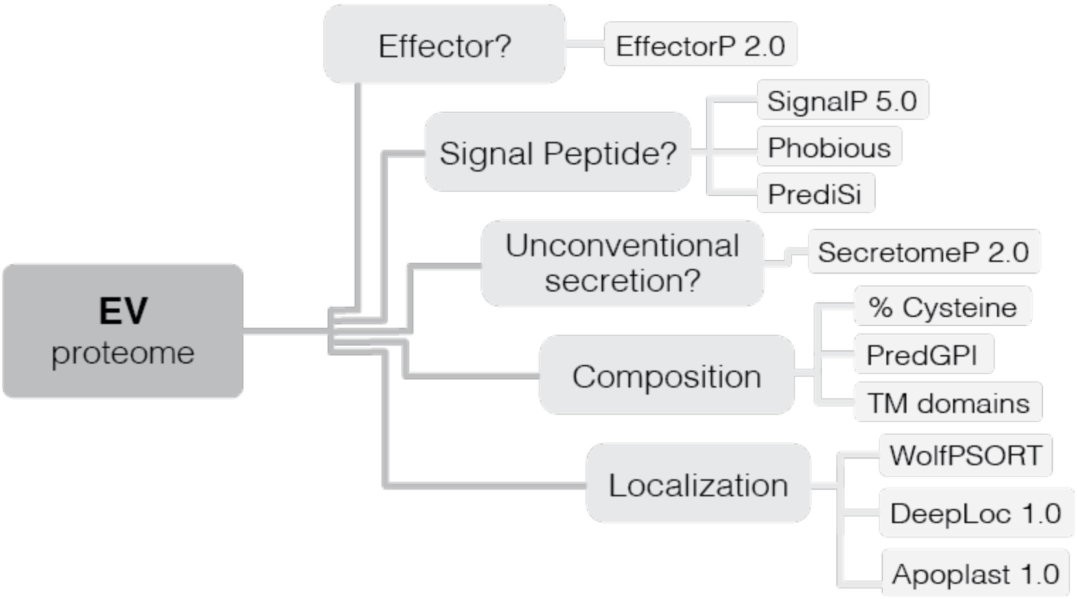

Supplementary Figure S4. Computational prediction of effector candidates detected in EV samples from *Fusarium graminearum* (*Fgr*). LFQ-based proteomics revealed 647 proteins in the EV samples from *Fgr*. All proteins were processed with EffectorP 2.0 to predict effector activity, PrediSi, Uniprot annotation, SignalP 5, and Phobius to predict signal peptide (SP), SecretomeP 2.0 to predict unconventional secretion, PredGPI to detect GPI anchoring, ApoplastP 1.0, WolfPSORT, and DeepLoc 1.0 to predict cellular location (ex: extracellular, cyt: cytoplasmic, mito: mitochondrial, nucl: nuclear). The percentage cysteine sequence content was calculated manually. Housekeeping, ribosomal and transmembrane (TM) proteins were omitted.
